# Effects of resistance training with/without Photobiomodulation on muscle and respiratory function in difficult-to-control asthma: a randomized trial

**DOI:** 10.1007/s10103-026-04880-x

**Published:** 2026-04-30

**Authors:** Ivan Peres Costa, Emilia Raposo Nascimento, Lawrence P. Cahalin, Etiene Farah Teixeira Carvalho, Edvane Aparecida Braz Araujo Silva, Roberto Stirbulov, Rodolfo Paula Vieira, Simone Dal Corso, Nivaldo Antônio Parizotto, Raquel Agnelli Mesquita-Ferrari, Luciana Maria Malosá Sampaio

**Affiliations:** 1 Universidade Nove de Julho, São Paulo, Brazil; 2 Department of Physical Therapy, University of Miami, Miami, USA; 3 Faculdade de Ciências Médicas da Santa Casa de São Paulo, São Paulo, Brazil; 4 Evangelica University of Goiás, Anápolis, Brazil; 5 Monash University, Melbourne, Australia; 6 Universidade Federal de São Carlos, São Carlos, Brazil

**Keywords:** Asthma, Resistance training, Photobiomodulation, Muscle strength, Respiratory function

## Abstract

Difficult-to-Control Asthma (DTCA) is characterized by persistent symptoms, frequent exacerbations, and reduced functional capacity despite optimized pharmacological therapy. Patients with DTCA commonly exhibit peripheral muscle dysfunction, making strategies that may potentiate the effects of resistance training clinically relevant. Photobiomodulation therapy (PBMT) has been investigated as a modality capable of improving muscle performance and exercise tolerance. The aim of this study was to determine whether combining resistance training with LED-based PBMT (RT+LEDT) results in greater improvements in peripheral muscle strength and functional exercise capacity compared with resistance training alone in adults with DTCA. This randomized, triple-blind controlled trial included 30 adults with DTCA who met predefined inclusion and exclusion criteria. Participants were allocated equally to an experimental group (RT+LEDT; n = 15) or a control group (RT; n = 15). Both groups completed supervised resistance training twice weekly for 12 weeks. The experimental group received active PBMT before each session, while the control group received placebo PBMT. The primary outcome was peripheral muscle strength assessed by one-repetition maximum (1RM). Secondary outcomes included cardiopulmonary exercise test variables, shuttle walk test performance, spirometry, physical activity level, and asthma control. Post-intervention group comparisons were performed using independent t-tests. Compared with RT alone, RT+LEDT produced greater post-treatment gains in 1RM strength across major muscle groups, along with significant improvements in oxygen consumption at the anaerobic threshold and shuttle walk test distance. No between-group differences were observed in pulmonary function or asthma control. PBMT combined with resistance training yielded clinically meaningful improvements in muscle strength and functional capacity, suggesting its potential as an adjunctive strategy in rehabilitation programs for DTCA.

**Supplementary Information:**

The online version contains supplementary material available at 10.1007/s10103-026-04880-x.

## Introduction

Asthma is a chronic inflammatory disease of the airways characterized by variable airflow limitation, bronchial hyperresponsiveness, and symptoms such as wheezing, dyspnea, chest tightness, and cough. It affects over 300 million individuals worldwide and represents a major cause of global morbidity, reduced quality of life, and considerable healthcare utilization [1]. Difficult-to-Control Asthma (DTCA) is a severe phenotype in which symptoms persist despite optimized pharmacologic therapy and adequate adherence, often leading to frequent exacerbations, chronic airway inflammation, and systemic consequences from prolonged corticosteroid use [2–4]. Beyond respiratory symptoms, patients with DTCA frequently develop peripheral muscle dysfunction, which contributes to exercise intolerance, early fatigue, and reduced functional capacity [5, 6] .

Resistance training is widely recognized as an essential component of pulmonary rehabilitation due to its capacity to improve skeletal muscle strength, enhance neuromuscular efficiency, and counteract functional limitations associated with chronic respiratory diseases. Evidence in patients with chronic obstructive pulmonary disease (COPD) demonstrates that structured resistance training leads to significant gains in muscle performance and daily functional abilities [7]. Given the similarity in peripheral muscle impairment observed in DTCA, exploring strategies that may boost the effects of resistance training is clinically important [8–12].

Photobiomodulation therapy (PBMT) is a noninvasive modality that employs non-ionizing light sources, such as low-intensity lasers or light-emitting diodes (LEDs). The light emitted during PBMT is absorbed by mitochondrial chromophores, particularly cytochrome c oxidase, leading to modulation of cellular metabolism. This photobiological interaction enhances mitochondrial function, increases adenosine triphosphate (ATP) synthesis, and promotes nitric oxide release. These mechanisms contribute to improved microcirculation and tissue oxygenation, which may enhance muscle energetics and delay the onset of fatigue. Importantly, PBMT is considered a safe intervention, as no clinically relevant adverse events or side effects have been described when it is applied within recommended parameters [13, 14]. Systematic reviews and meta-analyses indicate that PBMT applied before exercise can enhance performance and recovery in healthy adults and athletes [15]. However, the role of PBMT in respiratory disease populations remains under investigation, and most studies to date have focused on acute applications rather than long-term integration with structured training programs.

Although resistance training is an established component of pulmonary rehabilitation, evidence regarding its effects specifically in asthma - particularly in patients with DTCA - is still limited. Only a small number of studies have examined muscle performance outcomes in asthma populations, and most of them have focused on aerobic training rather than resistance-based protocols [16]. Likewise, research on photobiomodulation therapy in asthma remains sparse. Acute LED-based PBMT was evaluated by Costa et al. [17], who reported no improvement in fatigue time following a single application in adults with asthma, suggesting that isolated exposures may be insufficient to induce physiological adaptations. Recent reviews also emphasize that long-term PBMT protocols have not been systematically tested in asthma, despite promising biological mechanisms and preliminary findings in respiratory diseases [18], such as COPD [19]. This combination of limited clinical evidence, lack of long-term interventional trials, and absence of studies integrating PBMT with structured resistance training in asthma underscores a clear research gap. Addressing this gap is essential, as patients with DTCA frequently exhibit peripheral muscle dysfunction and reduced physical capacity, and may benefit from adjunctive strategies that enhance training-induced adaptations.

Considering the high prevalence of peripheral muscle dysfunction in DTCA and the potential of PBMT to augment physiological adaptations to exercise, investigating the combined effects of resistance training and LED-based PBMT may provide clinically relevant insights. The present study therefore examines whether LED-based PBMT delivered prior to resistance training enhances muscle strength and functional exercise capacity in adults with difficult-to-control asthma.

### Objective

The present study aimed to investigate the effects of resistance training combined with LED-based PBMT on peripheral muscle strength and functional exercise capacity in patients with difficult-to-control asthma. We hypothesized that the combination of resistance training with active PBMT would result in superior improvements compared with resistance training combined with inactive PBMT.

## Materials and methods

### Study design

We performed a prospective, randomized, triple-blind controlled trial conducted between February 2013 and December 2015, with all recruitment, baseline assessments, intervention sessions, and post-intervention evaluations completed within this period. The study was approved by the Research Ethics Committee of University Nove de Julho (Approval number: 1.691.019. All participants (or their legal guardians) provided written informed consent to participate in this study. All procedures performed in studies involving human participants were in accordance with the ethical standards of the institutional and/or national research committee and with the 1964 Helsinki declaration and its later amendments or comparable ethical standards. This study was registered in the ClinicalTrials.gov under the code NCT03112239 (date registration 2014-03).

### Participants, inclusion/exclusion criteria

**Fig. 1:**
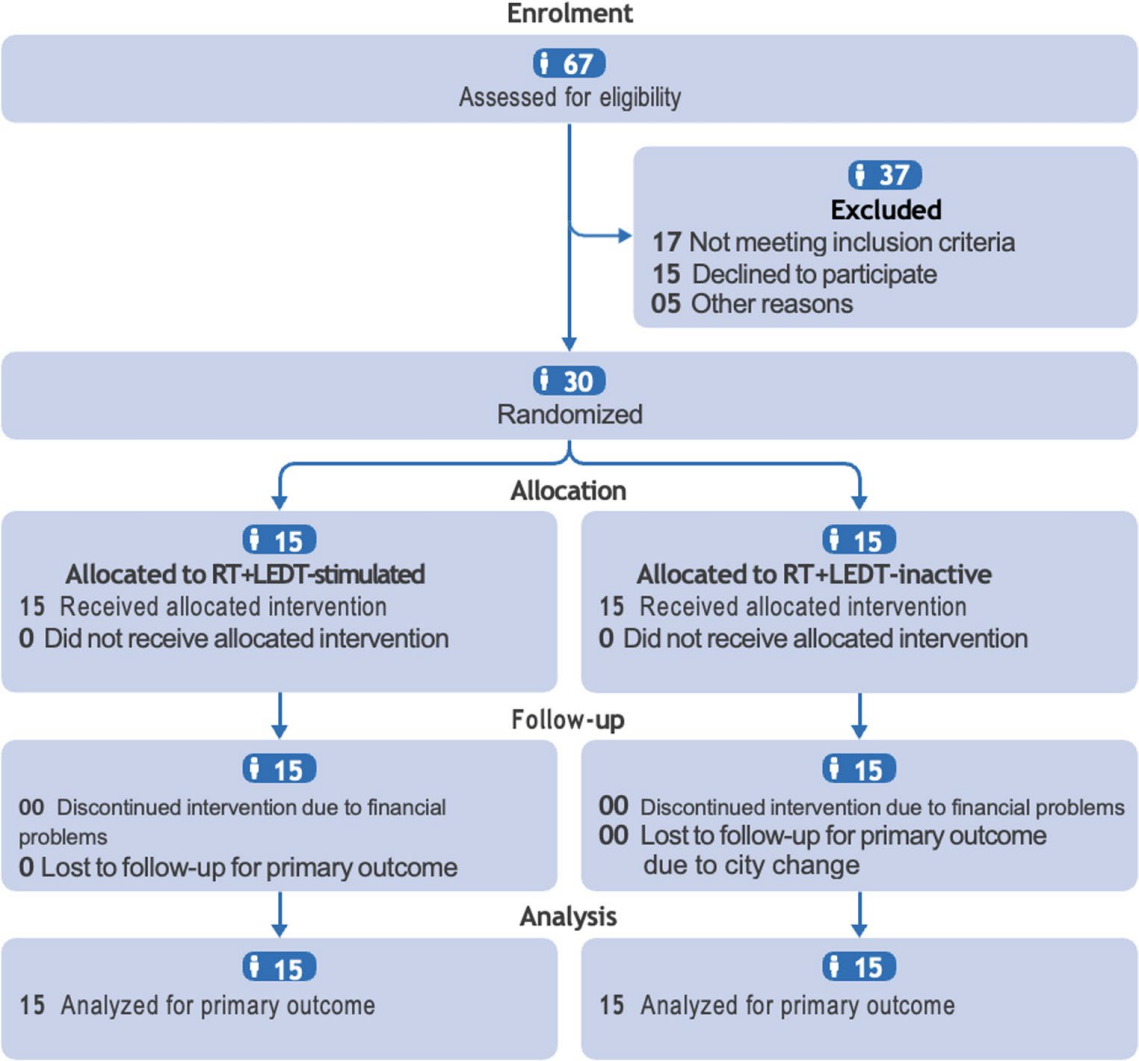
CONSORT 2025 Flow diagram. Flow diagram illustrating the progress through the phases of the randomized trial involving two groups, including enrollment, allocation, follow-up, and data analysis. CONSORT = Consolidated standards of reporting trials [23].

Participants were adults aged 20–70 years with a confirmed diagnosis of difficult-to-control asthma (DTCA), consecutively recruited from the outpatient asthma clinic of Santa Casa de Misericórdia de São Paulo. DTCA was defined according to established guideline criteria, including persistent symptoms despite optimized high-dose inhaled corticosteroids and long-acting bronchodilators, a history of frequent exacerbations (≥ 2 in the previous year or ≥ 1 hospitalization), and documented variable airflow limitation, in accordance with GINA and ERS/ATS definitions [9, 20]. Eligible participants were required to be in a clinically stable condition, characterized by the absence of exacerbations requiring systemic corticosteroids for at least three months, no respiratory infections in the preceding four weeks, and no changes in maintenance asthma therapy for at least six months, consistent with recommendations for exercise-based interventions in asthma [21]. Exclusion criteria included any condition that could interfere with exercise performance or compromise safety: cardiovascular or pulmonary comorbidities other than asthma; rheumatic, orthopedic, muscular, or cognitive impairments; pulmonary hypertension; uncontrolled systemic hypertension or diabetes; calcium-metabolism disorders; BMI ≥ 30 kg/m²; or contraindications to photobiomodulation therapy. Contraindications to PBMT were defined according to current safety recommendations and included photosensitivity or use of photosensitizing medications; active malignancy in the irradiation area; pregnancy involving abdominal or pelvic exposure; uncontrolled endocrine or metabolic disease; open wounds, active skin infections, or dermatitis at the treatment site; epilepsy precipitated by light exposure; and any other condition judged to pose risk or interfere with treatment response [20–22]. Photobiomodulation is considered a safe therapy with no reported adverse effects when applied within recommended parameters. Participants who met the inclusion and exclusion criteria were referred to Universidade Nove de Julho and included in the study (Fig. 1 – flowchart) .All participants were required to provide written informed consent, to participate in this study.

### Randomization & blinding procedures

Randomization was performed using a 1:1 allocation ratio generated by an independent researcher using random.org. Allocation concealment was ensured with sequentially numbered, opaque, sealed envelopes.

This study followed a triple-blind design.

- Participants were blinded to PBMT allocation, as the device emits no heat, sound, or visible cues that could reveal whether active or inactive irradiation was delivered.
- Outcome assessors responsible for all evaluations were blinded to group assignment throughout baseline and post-intervention assessments.
- The statistician and investigators responsible for data processing, statistical modeling, and interpretation were also blinded to group allocation until all analyses had been completed.

The PBMT devices were pre-programmed by an independent technician who had no role in recruitment, training supervision, outcome assessment, or statistical analysis. This ensured that all individuals interacting with participants or handling data (including assessors and statistical analysts) remained unaware of group allocation, fully characterizing a triple-blind methodology.

### Sample size

Based on previously published group mean one-repetition maximum (1RM) data reported by Dourado et al. [22] (33 ± 13 kg vs. 46 ± 11 kg), the expected between-group difference and standard deviations were used to estimate the effect size for the primary outcome. An a priori sample size calculation for a two-tailed independent-samples comparison of means, with a significance level of α = 0.05 and statistical power of 95%, indicated that a minimum of 14 participants per group would be required. To account for an anticipated dropout rate of approximately 10%, we planned to include 15 participants in each group. The sample size calculation was performed using the sample size module of SPSS, version 20 (IBM Corp., Armonk, NY, USA).

### Evaluations and outcome measures

#### Initial Assessments

Baseline assessments were performed before the start of the intervention and included primary and secondary outcome measures. All baseline and post-intervention evaluations were performed by the same licensed physiotherapists trained in cardiopulmonary and functional assessment, who were blinded to group allocation and not involved in the PBMT application, followed standardized protocols for each measure.

### Primary outcome

Peripheral muscle strength was assessed using the one-repetition maximum (1RM) test for the main muscle groups trained (quadriceps femoris, hamstrings, pectoralis major, latissimus dorsi, biceps brachii, and triceps brachii). After a specific warm-up set with light loads, resistance was progressively increased until the participant was able to complete only one full repetition with proper technique; up to three attempts were allowed per exercise, with 2–3 min of rest between attempts. This protocol follows international recommendations for strength assessment and has demonstrated excellent test–retest reliability (intraclass correlation coefficients > 0.90) in adults and in patients with chronic respiratory diseases [24]. This measure was chosen as the primary outcome because it directly reflects neuromuscular adaptations to resistance training [23]. SpO₂ and heart rate were monitored during tests. Blood pressure was measured before and after each test. Fatigue and dyspnea were assessed with the Borg scale [25].

## Secondary outcomes

### Cardiorespiratory fitness - cardiopulmonary exercise test (CPET)

Functional exercise capacity was evaluated using a cardiopulmonary exercise test (CPET) performed on a cycle ergometer (LODE BV, Corival) linked to a Breeze CardiO2 system (Medical Graphics Corp.) with a progressive, symptom-limited protocol (load increases, 5–15 watts/min, were customized) to reach volitional exhaustion in 8–12 min. Breath-by-breath gas exchange was recorded, and VO‚ÇÇpeak, oxygen consumption at the anaerobic threshold, ventilation, and respiratory exchange ratio were derived according to ATS/ACCP guidelines [26]. CPET variables show high reliability in chronic respiratory populations, with intraclass correlation coefficients typically above 0.85 for VO‚ÇÇpeak and anaerobic threshold [23]. ECG, HR, SpO₂, and BP were recorded. Dyspnea and fatigue were assessed via modified Borg scale [25]. Tests stopped for adverse symptoms or clinical signs.

### Functional exercise capacity – incremental shuttle walk test (ISWT)

Functional capacity was measured using the Incremental Shuttle Walk Test in a 10-m corridor, following the original Singh protocol [27]. Participants were instructed to walk back and forth between two cones at a pace dictated by standardized audio signals, with speed progressively increasing from 1.8 to 8.5 km/h over 12 incremental levels until symptom limitation or inability to maintain the required pace. Perceived exertion was monitored using the Borg scale [25]. Two ISWTs were performed on the same day, separated by a 20-minute rest period, and the longest distance achieved was used for analysis. The ISWT has demonstrated excellent test–retest reliability in patients with asthma, as reported by Costa et al. (2016) [28], with intraclass correlation coefficients exceeding 0.90.

### Pulmonary function - spirometry

Pulmonary function was assessed by spirometry CPFS/D USB spirometer, Medical Graphics^®^), assessing FVC, FEV₁, FEV₁/FVC according to ATS/ERS [29] and national guidelines [30], using at least three acceptable maneuvers and requiring reproducible values within accepted variability limits. Spirometric indices present high short-term reproducibility in stable asthma when standardized procedures are followed.

### Physical activity level – IPAQ (short form)

Habitual physical activity was assessed using the short version of the International Physical Activity Questionnaire (IPAQ-short), which estimates weekly energy expenditure in MET-minutes and classifies individuals as sedentary, active, or very active. The IPAQ-short has been validated in adults and demonstrates acceptable reliability (test–retest ICC ~ 0.75–0.80) [26].

### Asthma control – ACQ-6

Asthma control was evaluated using the six-item Asthma Control Questionnaire (ACQ-6), a validated instrument that assesses symptoms and rescue medication use over the previous week. The ACQ-6 score is calculated as the mean of the six items (0–6), with higher scores indicating poorer control; values ≥ 1.5 indicate uncontrolled asthma [31]. The ACQ-6 has demonstrated high internal consistency (Cronbach’s α > 0.90) and good test–retest reliability in adults with asthma, including Brazilian samples [32].

### Anthropometric measurements

Anthropometric measurements (weight, height, and BMI) and body composition assessed by bioelectrical impedance were obtained using standardized equipment, following manufacturer guidelines and performed under controlled conditions of hydration and time of day [33].

### Groups and interventions

Participants were randomly allocated into two parallel groups. Both groups completed a 12-week supervised resistance training program, performed twice weekly in the university exercise laboratory. Sessions were supervised by licensed physiotherapists not involved in evaluations.

### Resistance training procedures

The resistance training program targeted the major upper- and lower-limb muscle groups involved in functional activities and commonly affected in patients with chronic respiratory diseases. Exercises were performed using weight machines and free-weight equipment, with standardized positioning to ensure safety and reproducibility (details presented in Table 1).

**Table 1:** Resistance training protocol variables

Variable	Description
Training modality	Resistance training (machine-based exercises)
Muscle groups trained	Quadriceps femoris, hamstrings, latissimus dorsi, pectoralis major, biceps brachii, triceps brachii
Familiarization session	1 session prior to baseline testing
Training intensity	60–80% of one-repetition maximum (1RM)
Sets per exercise	2–3 sets
Repetitions per set	10 repetitions
Rest interval between sets	60–90 s
Rest between exercises	2–3 min
Load progression	Weekly increase of ~ 5%, according to tolerance
Training frequency	2 sessions per week
Session duration	Approximately 60 min
Total intervention duration	12 weeks
Total number of sessions	24 sessions
Training supervision	Supervised by trained physical therapists
Safety monitoring	Heart rate, SpO₂, blood pressure, Borg scale

Before each resistance-training session, participants performed a 5-minute standardized warm-up consisting of light cycling on an ergometer at 30–40% of perceived exertion, followed by dynamic mobilization of the major joints involved in the exercises (hips, knees, shoulders, and elbows). This procedure was used to increase muscle temperature and prepare the neuromuscular system for strength training [23].

At the end of each session, a 3-5-minute cool-down was performed, consisting of slow walking and gentle stretching of the trained muscle groups (quadriceps, hamstrings, chest, back, biceps, and triceps), consistent with rehabilitation recommendations for individuals with chronic respiratory diseases [7, 34, 35]. These procedures were applied identically in both groups and supervised by the same physiotherapists who monitored exercise execution.

### Quadriceps femoris

Strengthened using the knee extension machine (extensor chair) and the leg press. Participants were positioned seated with 90° of knee flexion for the extensor chair, while leg press exercises were performed with approximately 90° of hip and knee flexion at the starting position. These exercises were selected because quadriceps weakness is strongly associated with reduced exercise capacity and early fatigue in chronic airway diseases [7].

### Hamstrings

Trained using the knee flexion machine (flexor chair) performed in a seated or prone position depending on machine configuration. Hamstring strengthening is relevant for gait mechanics and postural control [35].

### Pectoralis major

Trained using the barbell bench press (supino), performed lying supine with feet on the floor, shoulder blades retracted, and elbows positioned at approximately 45° abduction. This exercise efficiently targets horizontal shoulder adduction and upper-limb pushing strength [35].

### Latissimus dorsi

Trained using the lat pulldown machine, performed seated with knees stabilized under thigh pads, trunk upright, and hands placed at a shoulder-width grip [35].

### Biceps brachii

Strengthened using free-weight dumbbell curls performed seated or standing, with elbows close to the torso, or using cable pulley curls, allowing constant tension and strict movement patterns [35].

### Triceps brachii

Trained using a triceps cable extension performed standing with the elbows fixed to the torso, ensuring isolated elbow extension, or using overhead triceps extension variations adapted to participant tolerance [35].

All exercises were performed at 60–80% of 1RM, following standard recommendations for strength development in clinical and rehabilitation settings [7, 34], with 2–3 sets of 10 repetitions and 1–2 min of rest between sets, as suggested in established resistance-training guidelines.¹ Weekly 5% load progression was applied based on movement quality, tolerance, and 1RM reassessments, in accordance with recognized principles of progressive overload [7]. Total session duration ranged from 45 to 60 min, and all sessions were supervised by physiotherapists specialized in resistance training for chronic respiratory conditions [35].

Throughout the 12-week intervention, participants were actively monitored for potential adverse events related to both resistance training and PBMT. No side effects or complications were observed during exercise execution, warm-up, cool-down, or PBMT application. PBMT was well tolerated, with no reports of skin irritation, discomfort, burns, or post-session soreness, consistent with its established safety profile [34, 35]. Similarly, no musculoskeletal injuries, exacerbations, or exercise-induced respiratory symptoms occurred during resistance training sessions. All procedures were supervised by trained physiotherapists, and participants were instructed to report any symptoms immediately.

## Photobiomodulation Therapy

**Fig. 2:**
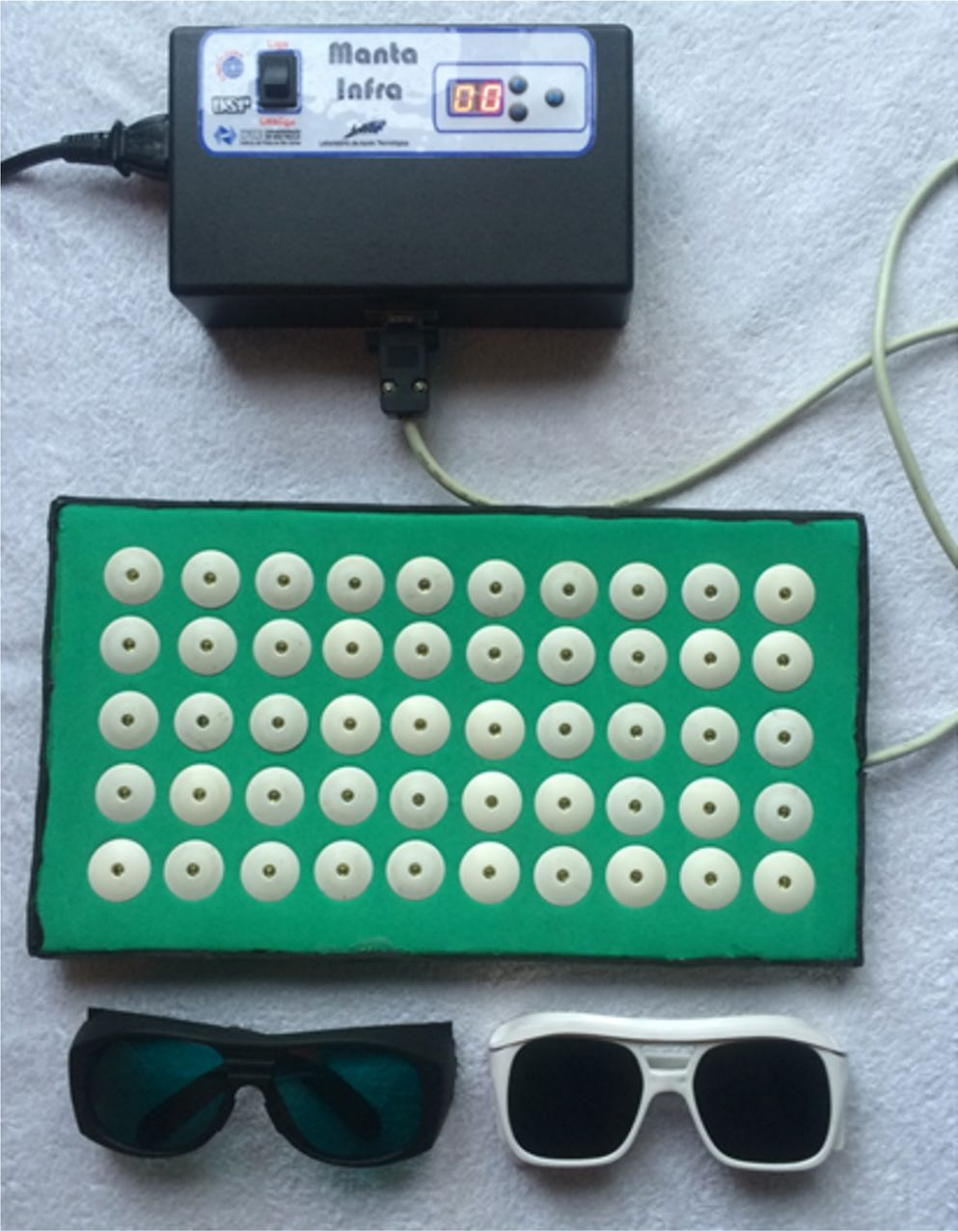
Photobiomodulation device consisting of 50 LEDs (λ = 850 ± 20 nm), custom-built by Universidade Federal de São Carlos and Universidade de São Paulo

PBMT (active or inactive) was administered immediately before each training session, using a custom-built 50-LED probe (λ = 850 ± 20 nm in continuous emission mode), developed by the Federal University of São Carlos and the University of São Paulo (Fig. 2). Calibration was performed using Thorlabs^®^ equipment. Each LED delivered an average power output of 50 mW, with an irradiance of 250 mW/cm² and a spot size of 0.2 cm². The irradiation time was 15 s per point, resulting in an energy delivery of 0.75 J per LED and a radiant exposure of 3.75 J/cm². A total of 50 points were irradiated per muscle group, corresponding to 37.5 J per muscle group and 262.5 J per session. Parameters were based on prior literature (Table 2) [36–38].

**Table 2:** Parameters of LEDT and regions of irradiation on muscle groups before resistance training protocol

Number of LED’s: 50;
**Wavelength**: 850 ± 20 nm (infrared);
**Frequency**: Continuous output;
**Average Power**: 50 mW per/LED;
**LED spot size**: 0.2 cm^2^;
**Irradiance**: 250 mW/cm^2^;
**Time exposure of each point**: 15 s;
**Energy per LED**: 0.75 J;
**Radiant exposure per LED**: 3.75 J/cm^2^;
**Number of irradiated points per muscle group**: 50;
**Total energy delivered per muscle group**: 37.5 J;
**Muscle groups irradiated before RT**: Femoral Quadriceps, Hamstrings, Large Dorsal, Pectoralis, Brachial Biceps and Brachial Triceps
**Total energy delivered per session on body**: 262.5 J;
**Total power output**: 2500 mW;
**Application mode**: device held coupled in skin contact.

Legends: *LEDT* light-emitting diode therapy, *LED* light-emitting diode, *J* Joule, *mW* milliwatt, *cm*^2^square centimeters,*nm* nanometers, *s* seconds

PBMT was applied in direct skin contact bilaterally to all muscle groups involved in the resistance training protocol (quadriceps femoris, hamstrings, tibialis anterior, latissimus dorsi, pectoralis major, biceps brachii, and triceps brachii), immediately prior to each training session, twice weekly for 12 weeks (24 sessions) as shown in Fig. 2. The dosimetric parameters (as shown in table 2) were selected based on prior experimental and clinical evidence demonstrating optimal tissue penetration, mitochondrial stimulation, and enhancement of muscle performance when PBMT is applied before exercise [36–41].

### Experimental group (RT+LEDT-active)

Participants in this group underwent the resistance training program combined with active PBMT. PBMT was delivered immediately before each training session using the LEDT device described above, with all parameters (wavelength, irradiance, energy per point, number of points, and total session dose) applied according to the predefined protocol.

### Control group (RT+LEDT-inactive)

Participants completed the same resistance training program; however, the PBMT device was applied with identical appearance, contact, timing, and procedures, but without emission of therapeutic light. This ensured a true placebo condition while maintaining triple blinding (participants, therapists, and assessors).

## Final evaluation

After 12 weeks, all baseline tests were repeated.

### Statistical analysis

The Shapiro-Wilk test was used to assess the normality of anthropometric, demographic, and clinical data. Parametric data are presented as means and standard deviations, while non-parametric data are expressed as medians and interquartile ranges. Lean mass, 1RM, cardiopulmonary exercise test variables (oxygen uptake - VO₂_max_ and anaerobic threshold), and Shuttle Walk Test distance (meters) were analyzed using linear regression models to evaluate the effects of LEDT on absolute delta, percentage delta, and baseline-adjusted changes. Linear regression models were selected a priori as the primary analytical approach because they allow adjustment for baseline values, improve statistical precision, and reduce bias associated with regression to the mean. Mixed MANOVA was not used because several outcomes did not meet its methodological assumptions (multivariate normality and homogeneity of covariance matrices), and the modest sample size (*n* = 15 per group) limits the stability of multivariate models. ANCOVA/regression-based analyses are widely recommended for randomized controlled trials with continuous outcomes, according to CONSORT and established methodological literature. A sensitivity analysis was performed using re-evaluation values, adjusted for each outcome’s initial value. The probability of a type I error was set at 0.05. All analyses were conducted using SPSS software, version 20 (IBM Corp., Armonk, NY, USA). Deidentified data collected and presented in this study, including individual participant data and a data dictionary defining each field in the set, will be made available upon reasonable request after publication of this Article, following approval by regulatory authorities. Data can be requested by contacting the corresponding author.

## Results

A total of 67 patients were screened for eligibility. Of these, 37 were excluded for the following reasons: not meeting inclusion criteria with cardiovascular comorbidities (*n* = 12) and orthopedic limitations (*n* = 5), declining participation after initial screening (*n* = 15), and inability to establish contact (*n* = 5) after their relocation to another city. The remaining 30 participants were randomized into the two study groups, completed the study schedule and were included in the final analyses: control group (RT+LEDT-inactive) (*n* = 15), and experimental group (RT+LEDT-stimulated) (*n* = 15).

Table 3 shows the anthropometric, demographic, and clinical characteristics of the two groups. The training protocol was performed safely, without any adverse events documented in any patient. Baseline characteristics of both groups were similar (age = 37.22 ± 10.44 Years Kg/m^2%^ and 36.7 ± 11.73; BMI = 26.70 ± 4.23 and 26.58 ± 5.72, pulmonary function = 66.0 ± 32.08 and 67.0 ± 31.90; and medication in use Long-acting β-2 agonist: 100%).

**Table 3:** Anthropometric, demographic and clinical data

Variables	RT+LEDT-active	RR+LEDT-inactive
	(*n* = 15)	(*n* = 15)
Age	37.22 ± 10.44	36.7 ± 11.73
Gender (M/F)	6/9	7/8
Height (m)	1.65 ± 0.87	1.60 ± 0.67
Weight (Kg)	72.76 ± 10.61	71.12 ± 12.03
BMI (Kg/m^2^)	26.70 ± 4.23	26.58 ± 5.72
FVC (%)	66.0 ± 32.08	67.0 ± 31.90
FEV_1_ (%predict)	55.0 ± 31.61	54.0 ± 32.52
ACQ	1.57 ± 0.91	1.59 ± 0.98
**IPAQ**,** n (%)**		
Inactive	6 (40%)	7 (46,66%)
Moderately Active	5 (33,33%)	6 (40%)
Very Active	4 (26,66%)	2 (13,33%)
**Medication in use**,** n (%)**		
Long-acting β-2 agonist	15 (100%)	15 (100%)

Data are expressed as mean ± standard deviation, median (interquartile range), number of participants and ratio in percentage. Legend: *RT* Resistance training, *LEDT* light-emitting diode therapy, *M* male, *F* female, *BMI* Body mass index, *FVC* Forced vital capacity; *FEV1* forced expiratory volume in the first second, *ACQ* Clinical asthma control questionnaire, *IPAQ* International questionnaire on physical activity (According to the IPAQ classification)

Table 4 presents the linear regression analysis of muscle mass and 1RM variables for the muscles targeted during RT (pectoralis major, latissimus dorsi, biceps brachii, triceps brachii, quadriceps femoris, and hamstrings). The analysis compared the experimental group with the control group, with results expressed as 95% confidence intervals (CI) based on the total sample values from the experimental group (unadjusted). Additionally, the results were adjusted for gender, and corresponding p-values are reported. While no statistically significant differences were found between groups for lean mass, significant improvements in 1RM values were observed across all trained muscle groups in the experimental group.

**Table 4:** Linear regression analysis of 1RM values for the muscles trained in the RT+LEDT-active group

Variables				
	Gross Β (95%IC)	*p*	Adjusted Β (95%IC)	*p*
**LM (Kg)**	1.87 (-0.55; 4.28)	0.12	-1.82 (-4.28;0.64)	0.142
**1-RM QD (Kg)**	32.9 (16.8; 49.1)	0.00	33.2 (16.7; 49.6)	0.001
**1-RM IT (Kg)**	22.5 (4.3; 40.7)	0.01	23.2 (4.8; 41.5)	0.012
**1-RM Pectoralis (Kg)**	27.6 (2.5; 52.6)	0.03	28.3 (2.8; 53.7)	0.034
**1-RM Bíceps (Kg)**	67.9 (19.6; 116.4)	0.00	69.8 (21.1; 118.5)	0.001
**1-RM Tríceps (Kg)**	19.3 (-2.3; 40.9)	0.07	20.7 (-0.3; 41.7)	0.055
**1-RM LD (Kg)**	29.2 (11.7; 46.7)	0.00	30.3 (13.2; 47.3)	0.001

Legend: *RT* Resistance Training, *LEDT* light-emitting diode therapy, *LM* Lean mass, *1-RM* 1 Repetition maximum, *QD* Quadriceps, *IT* Hamstrings, *LD* Latissimus Dorsi

In both gross and adjusted models, significant increases were observed in the Quadriceps (1-RM QD), hamstrings (1-RM IT), Pectoralis (1-RM Pectoralis), Biceps (1-RM Biceps), and Latissimus Dorsi (1-RM LD), with p-values ranging from 0.001 to 0.03. These results indicate that the combination of RT with LEDT contributed to significant strength improvements in these muscle groups.

In contrast, lean mass and triceps muscle strength did not demonstrate significant associations in either statistical model (*p* > 0.05), suggesting that LEDT did not have a relevant impact on these parameters.

When compared to the control group, these findings suggest that the active LEDT condition provided additional benefits in strength gains, particularly for the Quadriceps, Hamstrings, Pectoralis, Biceps, and Latissimus Dorsi muscles. This reinforces the potential of LEDT to enhance muscle-specific strength adaptations when combined with resistance training.

The linear regression analysis of functional capacity variables (VO_2_ AT, VO_2 max_, and Shuttle Walk Test distance - SWTDm) are presented in Table 5. The data are shown with 95% confidence intervals (CI) referring to the total sample of the experimental group (unadjusted). The values are also adjusted for sex, with p-values provided. The results revealed significant improvements in functional capacity variables in the experimental group. Both gross and adjusted models showed significant increases in VO₂ at the anaerobic threshold (VO₂ AT) (Gross: B = 30.8; 95% CI: 9.8–51.8; *p* = 0.004 / Adjusted: B = 31.0; 95% CI: 9.5–52.5; *p* = 0.005) and in the six-minute walk test distance (SWTD) (Gross: B = 18.8; 95% CI: 4.9–32.7; *p* = 0.01 / Adjusted: B = 16.8; 95% CI: 6.4–27.4; *p* = 0.003).

**Table 5:** Linear regression analysis of the functional capacity variables of the RT+LEDT-active group

Variables				
	Gross Β (95% IC)	*p*	Adjusted Β (95% IC)	*p*
**VO**_**2**_ **(AT) (ml/kg/min)**	30.8 (9.8; 51.8)	0.001	31.0 (9.5; 52.5)	0.001
**VO**_**2 máx**_	9.1 (-1.4; 19.7)	0.052	9.3 (-1.5; 20.1)	0.005
**SWTD (m)**	18.8 (4.9; 32.7)	0.012	16.8 (6.4; 27.4)	0.001

The data are presented in 95% IC compared to both groups and adjusted for gender. Legend: *RT* Resistance Training, *LEDT* light-emitting diode therapy, *VO*_2_Oxygen Consumption, *AT* Anaerobic Threshold, *VO*_2_ max Oxygen consumption at peak of exercise, *SWT* Shuttle walk test distance

For VO₂ peak, there was no significant association in the gross model (B = 9.1; 95% CI: -1.4 to 19.7; *p* = 0.09), but after adjustment, the result became marginally significant (B = 9.3; 95% CI: -1.5 to 20.1; *p* = 0.05), suggesting a possible trend toward improvement. These results indicate that RT combined with LEDT produced meaningful improvements in submaximal functional capacity (VO₂ AT and SWTD), with a tendency to improve VO₂ peak as well.

## Discussion

The present study demonstrated that combining resistance training with active LED-based photobiomodulation therapy (RT+LEDT) resulted in greater improvements in peripheral muscle strength and functional exercise capacity compared with resistance training alone. Participants in the experimental group showed superior gains across multiple muscle groups, higher oxygen consumption at the anaerobic threshold, and better performance in submaximal functional tests. These findings suggest that the addition of photobiomodulation therapy may enhance training adaptations in patients with difficult-to-control asthma.

Prior to this study, although resistance training was known to produce meaningful strength gains in individuals with chronic respiratory diseases such as COPD [7], its combined use with photobiomodulation therapy in asthma had not been explored. Evidence from healthy individuals [39–41] and athletes [38, 40] indicates that PBMT can enhance contractile function and facilitate recovery, supporting its integration into structured training protocols. Our findings of significant strength gains in the experimental group reinforce this potential synergistic effect.

These results differ from findings of Costa et al. [17] who reported no improvement in fatigue time after a single LEDT session in patients with asthma. Their null findings may relate to the acute application, insufficient irradiation dose, or limited treatment area [41], underscoring that isolated exposures are unlikely to induce meaningful adaptations. By contrast, the present study implemented a long-term protocol with repeated pre-training PBMT, which appears more compatible with neuromuscular adaptation processes. Previous experimental studies have shown that repeated PBMT sessions are required to induce sustained mitochondrial and microvascular adaptations, which likely explains the discrepant findings between acute and chronic protocols. The magnitude of strength gains observed in the present study is consistent with the expected response to resistance training in chronic respiratory diseases, as demonstrated by Panton et al. [42], who reported a 36% increase in muscle strength following a structured resistance training program in patients with COPD. Similar findings have also been reported in studies investigating the adjunctive use of photobiomodulation. Baroni et al. [43], for example, demonstrated that low-level laser therapy (LLLT; wavelength 808 nm) applied before eccentric knee extensor training enhanced strength gains compared with training alone (20.5% vs. 13.7%). The use of near-infrared wavelengths in these studies is consistent with the parameters adopted in the present protocol (850 ± 20 nm LED-based photobiomodulation), which are known to provide deeper tissue penetration and effective stimulation of mitochondrial chromophores involved in muscle bioenergetics.

The physiological rationale for PBMT as an adjunct to resistance training is primarily related to its effects on skeletal muscle bioenergetics and microcirculation. Infrared light in the 800–900 nm range penetrates deeply into muscle tissue and is absorbed by mitochondrial chromophores, particularly cytochrome c oxidase, leading to increased electron transport chain activity and enhanced adenosine triphosphate (ATP) synthesis [44, 45]. This mechanism improves muscle energy availability, delays fatigue onset, and enhances contractile efficiency during exercise. Additionally, PBMT has been shown to modulate nitric oxide (NO) signaling, promoting vasodilation and improving local blood flow and oxygen delivery to active muscles [46]. These combined effects provide a mechanistic basis not only for strength gains, but also for improvements in submaximal exercise tolerance observed in the present study. Collectively, these mechanisms provide a biologically plausible explanation for the superior gains in muscle strength and submaximal functional capacity observed in the experimental group.

Functional capacity also improved significantly in the experimental group, consistent with previous studies showing that PBMT may accelerate VO₂ kinetics and improve exercise tolerance. Ferraresi et al. [38] reported improved VO₂ responses in an elite athlete after LEDT, while Miranda et al. [47] demonstrated increased distance and exhaustion time during cardiopulmonary exercise in untrained males. These effects are thought to be mediated by enhanced peripheral oxygen utilization, improved mitochondrial efficiency, and better matching between oxygen delivery and metabolic demand. Dourado et al. [22] found that resistance training alone improved six-minute walk distance in COPD, though broader functional domains remained unchanged, indicating that adjunctive therapies may be required for more comprehensive benefits. Our findings of increased VO₂ max differ from those of Vonbank et al. [48], who observed improvements only in endurance or combined training modalities, suggesting that PBMT may help overcome some limitations of resistance-only protocols.

No between-group differences were observed in body composition after the 12-week intervention. This finding is consistent with prior evidence showing that short-term resistance training often elicits predominantly neural adaptations, whereas measurable hypertrophic changes typically require longer training duration, higher training volume, or higher weekly Frequency. Although PBMT has been shown to modulate muscle metabolism and reduce oxidative stress, its effects on muscle hypertrophy appear to depend on prolonged exposure and higher training volumes. Additionally, pulmonary rehabilitation guidelines highlight that improvements in functional capacity may occur without significant alterations in lean mass in respiratory populations [7]. These observations suggest that the absence of detectable changes in body composition in our sample is expected and does not contradict the strength gains observed.

Physical activity level assessed by the IPAQ remained unchanged in both groups. This pattern is aligned with literature showing that improvements derived from supervised exercise programs do not automatically translate into changes in habitual daily activity without behavioral interventions [7, 49, 50]. Daily physical activity behavior is influenced by psychosocial and environmental factors, which are not directly modified by physiological conditioning alone. The ATS/ERS Statement similarly reports that pulmonary rehabilitation enhances exercise tolerance but does not consistently modify free-living physical activity levels [25]. Thus, the stable IPAQ scores observed in our study likely reflect that the intervention targeted physical capacity rather than behavioral modification.

Asthma control remained stable across both groups, with no significant between-group differences. This result is consistent with systematic reviews showing that exercise interventions primarily promote peripheral and functional adaptations, with minimal short-term impact on asthma symptoms or airway inflammation [50]. Because neither resistance training nor PBMT directly targets airway inflammation or bronchial hyperresponsiveness, changes in asthma control scores were not expected. The Cochrane Review on physical training for asthma likewise reports that exercise improves fitness but does not produce clinically meaningful changes in asthma control scores [50]. These findings reinforce that the enhanced functional outcomes observed in our RT+PBMT group were driven by muscular and metabolic adaptations rather than direct effects on disease control.

Spirometric variables showed no significant changes after the intervention, a finding consistent with the broader literature. The Cochrane review on exercise in asthma reports that physical training does not meaningfully alter forced expiratory volumes in adults with stable disease [50]. Likewise, the GINA 2024 update emphasizes that non-pharmacological interventions-such as exercise training do not modify the underlying airway pathology or baseline lung function in asthma [1]. These findings support the interpretation that improvements observed in the present study occurred independently of changes in ventilatory mechanics.

Some studies have reported inconsistent findings regarding PBMT. Toma et al. [51] found no additional strength benefits from combining LLLT with RT in elderly women, an outcome potentially explained by population differences or PBMT parameters. Conversely, Bublitz et al. [52] demonstrated that LLLT reduced perceived lower-limb fatigue in heart failure patients, reinforcing PBMT’s capacity to influence exercise responses even in populations with cardiometabolic limitations. Together, these studies highlight that PBMT effects are context-dependent and strongly influenced by protocol characteristics. Despite heterogeneity studies, the present findings suggest that PBMT, when applied consistently and with appropriate dosimetry, may augment training-induced adaptations in asthma.

To our knowledge, this is the first study to evaluate the combined effects of resistance training and LED-based PBMT on muscle strength and functional performance in patients with asthma. Pulmonary rehabilitation is a core component of asthma management, and adjunctive therapies such as PBMT hold potential to enhance exercise capacity in individuals who often present with peripheral muscle dysfunction and exertional intolerance [7]. Given its non-invasive nature and favorable safety profile, PBMT represents a promising addition to comprehensive rehabilitation strategies.

The combination of PBMT with resistance training offers several advantages, including its non-invasive nature, absence of systemic adverse effects, and ability to potentiate training adaptations without increasing mechanical load. This is particularly relevant in patients with DTCA, who frequently exhibit peripheral muscle dysfunction and exercise intolerance [6]. 

Notably, PBMT efficacy is highly dependent on dosimetry. Key parameters—such as wavelength, irradiance, total energy, treatment area, and timing—strongly influence therapeutic outcomes. Inadequate or acute dosing can fail to produce physiological effects, as demonstrated in previous negative trials [17]. The present study employed a pre-exercise PBMT protocol aligned with parameters supported by controlled trials and systematic reviews [15, 37], which may have contributed to the observed additive benefits. These considerations highlight the importance of standardized reporting and careful dosimetric control to enable reproducibility and clinical translation.

### Study limitations

This study has some limitations that should be acknowledged. First, the relatively small sample size may limit the generalizability of the findings, despite being adequately powered to detect differences in the primary outcome. Second, the absence of a PBMT-only group prevents isolation of the independent effects of photobiomodulation therapy, which may be relevant for patients unable to perform exercise, such as those with severe disease or steroid-induced myopathy. Third, the lack of long-term follow-up precludes evaluation of the durability of the observed adaptations after cessation of the intervention. In addition, biochemical markers of muscle damage, inflammation, and oxidative stress were not assessed, limiting mechanistic interpretation. The absence of surface electromyography restricted objective analysis of neuromuscular fatigue, and imaging techniques such as ultrasound were not used to quantify morphological adaptations, including muscle hypertrophy. Finally, habitual physical activity was assessed by self-report, which may be subject to recall bias. Despite these limitations, the randomized, controlled, and triple-blinded design and the consistency of the primary outcomes support the validity of the findings.

Future studies should include larger and more diverse samples to improve external validity and explore whether the observed benefits are consistent across different asthma phenotypes and severity levels. Randomized trials incorporating a PBMT-only group and longer follow-up periods are warranted to clarify the independent and sustained effects of photobiomodulation therapy. Additionally, the inclusion of biochemical markers of muscle metabolism, oxidative stress, and inflammation, as well as neuromuscular and imaging assessments, would allow deeper mechanistic insight. Finally, future investigations should evaluate different PBMT dosimetric parameters and integrate behavioral strategies to determine whether physiological gains translate into long-term improvements in physical activity and clinical outcomes.

## Conclusion

Resistance training alone improved peripheral muscle strength in patients with difficult-to-control asthma; however, the addition of LED-based photobiomodulation therapy produced superior and clinically meaningful gains. The combined intervention resulted in greater improvements not only in muscle strength but also in functional exercise capacity, as demonstrated by higher oxygen consumption at the anaerobic threshold, increased peak oxygen uptake, and longer shuttle walk test distances. These findings highlight photobiomodulation therapy as a promising adjunct to resistance training, with potential to optimize rehabilitation outcomes in this population. Further studies are warranted to confirm these benefits and to refine photobiomodulation protocols for long-term clinical application.

1. Clinical trial number: NCT03112239.
2. Ethics statement: (CoEP-UNINOVE), São Paulo, Brazil (under protocol No. 1.691.019).
3. Patient consent statement: (CoEP-UNINOVE), São Paulo, Brazil (under protocol No. 1.691.019).
4. Committe de Ethics Universidade Nove de Julho – number 1.691.019.

## Supplementary Information

Below is the link to the electronic supplementary material.

Supplementary Material 1

## Data Availability

Deidentified data collected and presented in this study, including individual participant data and a data dictionary defining each field in the set, will be made available upon reasonable request after publication of this Article, following approval by regulatory authorities. Data can be requested by contacting the corresponding author.
